# Clinical correlation of nuclear survivin in esophageal squamous cell carcinoma

**DOI:** 10.1007/s12032-012-0225-9

**Published:** 2012-04-19

**Authors:** Marco K. C. Hui, Kenneth K. Y. Lai, Kwok Wah Chan, John M. Luk, Nikki P. Lee, Yvonne Chung, Leo C. M. Cheung, Gopesh Srivastava, Sai Wah Tsao, Johnny C. Tang, Simon Law

**Affiliations:** 1 Department of Surgery, Queen Mary Hospital, The University of Hong Kong, Pokfulam, Hong Kong, China; 2 Department of Pathology, The University of Hong Kong, Hong Kong, China; 3 Department of Oncology, Roche R&D Center, pRED China, Shanghai, China; 4 Department of Anatomy, The University of Hong Kong, Hong Kong, China; 5 Department of Applied Biology and Chemical Technology, Hong Kong Polytechnic University, Hong Kong, China

**Keywords:** Esophageal squamous cell carcinoma, Nuclear survivin, Nodal metastasis, Pathological stage, Biomarker

## Abstract

To examine the correlation of survivin (both total and nuclear survivin) with clinicopathological parameters of esophageal squamous cell carcinoma (ESCC) patients. Tumors and non-tumor tissues near the proximal resection margins were resected from ESCC patients undergone esophagectomy. Quantitative polymerase chain reaction (qPCR) was performed to detect survivin mRNA expression level in the 10 paired tumor and adjacent non-tumor tissues. To confirm with the clinical situation, survivin mRNA and protein expression were measured by qPCR and immunoblot, respectively, in 5 ESCC cell lines and a non-neoplastic esophageal epithelial cell line. Immunohistochemistry was employed to reveal the cellular localization of survivin in tumor tissues isolated from the 64 ESCC patients undergone surgery alone. Up-regulation of survivin mRNA and protein was found in 5 ESCC lines (HKESC-1, HKESC-2, HKESC-3, HKESC-4, and SLMT-1) when compared to a non-neoplastic esophageal epithelial cell line NE-1. In particular, HKESC-3, HKESC-4, and SLMT-1 cells demonstrated ~50-fold increase in survivin mRNA. High level of survivin mRNA in tumor tissues when compared to non-tumor tissues was found in 70 % (7 of 10) of clinical cases. The increase in expression ranged from ~twofold to ~16-fold. Immunohistochemistry results showed that survivin was found at the cell nuclei in all specimens examined. Nuclear expression of survivin was inversely associated with the likelihood of developing nodal metastasis (*p* = 0.021) and significantly associated with early-stage ESCC (*p* = 0.039). Nuclear survivin could serve as a marker for indicating disease status in ESCC patients.

## Introduction

Esophageal cancer is the eighth most prevalent cancer worldwide and ranks sixth as the most common cause of cancer-related deaths. Among all histological subtypes, esophageal squamous cell carcinoma (ESCC) is the predominant type in Asia. Unfortunately, the prognosis of ESCC is relatively poor and survival rate is low [1]. Recently, increasing number of studies has been dedicated to identify important prognostic markers for this malignancy, for which survivin is a potential one [2–8].

Survivin is a 16.5-kDa protein with 142 amino acid residues. It is reported to be highly expressed in many types of cancers but rarely present in normal tissues [9]. Survivin is one of the key players in the complex network regulating apoptosis. It belongs to the family of the inhibitor of apoptosis proteins (IAPs), which is a group of proteins that counteract programmed cell death through inhibiting caspase 3, 7, and 9 [10]. Five isoforms of survivin arisen from alternative splicing have been identified so far [11–14]. Among them, the wild-type survivin is the most studied isoform and is shown to involve in progression of various cancers [15–17].

Based on the serial analysis of gene expression (SAGE) study, survivin is proposed to be a promising prognostic biomarker for tumor [18]. The prognostic value of survivin has been reported in several studies of different cancer types [9]. By using biochemical methods of immunohistochemistry or subcellular fractionation, survivin can be detected in cell nucleus and cytoplasm and that these two pools of survivin are indeed functionally different [12].

Conflicting data exist regarding the cellular localization of survivin. While most immunohistochemical studies reporting the cytoplasmic localization of survivin [9], some demonstrated the localization of survivin in the nucleus and correlated with better prognosis [19, 20]. Still, limited information is available for stating the cellular localization of survivin in ESCC. Therefore, it is of interest to investigate the cellular localization of survivin and its potential clinical significance in ESCC patients.

## Materials and methods

### Human cell lines

5 ESCC lines (HKESC-1, HKESC-2, HKESC-3, HKESC-4, and SLMT-1) and a non-neoplastic esophageal epithelial cell line NE-1 used in this study were established by our team earlier [21–25]. The culture condition of these cell lines was followed as described previously. NE-1 cells were cultured in Keratinocyte-SFM medium (Invitrogen, Carlsbad, CA). HKESC-1 and HKESC-4 were maintained in minimum essential medium (MEM) medium (Invitrogen) with 10 % fetal bovine serum (FBS), while HKESC-2, HKESC-3, and SLMT-1 were cultured in MEM medium with 20 % FBS. All types of medium used in this study were supplemented with 1 % penicillin–streptomycin. Cultures were incubated at 37 °C in a humidified atmosphere containing 5 % CO_2_.

### Clinical specimens

Sixty-four ESCC patients undergone esophagectomy between 1997 and 2005 at Queen Mary Hospital, Hong Kong, were recruited for this study, and the patient data were summarized in Table 1. None of the patients received neoadjuvant chemotherapy or radiotherapy. Consent regarding the use of clinical specimens for this study was obtained from Institutional Review Board of The University of Hong Kong/Hospital Authority Hong Kong West Cluster (HKU/HA HKW IRB). Tumors and non-tumor tissues near the proximal resection margins were resected. A portion of the specimens was processed for routine histopathological diagnosis by fixing in 10 % formalin followed by embedding in paraffin; 5 μm sections were prepared and stained with hematoxylin and eosin for light microscopy. These sections were examined and classified into different grades based on the criteria of the World Health Organization. A portion of the remaining specimens was then frozen for long-term storage until use. The median follow-up period of the patients is 14.1 months (ranged from 1.2 to 90.6 months).

**Table 1:** Clinical and pathological parameters of esophageal squamous cell carcinoma patients

	Number of cases	%	Median (range)
Age (years)	64		65 (40–87)
Gender			
Female	17	26.6	
Male	47	73.4	
Smoking			
Non Smoker	31	48.4	
Smoker	33	51.6	
Level of tumor			
Upper	10	15.6	
Middle	37	57.8	
Lower	14	21.9	
Double	3	4.7	
Tumor differentiation			
Poor	14	21.9	
Moderate	38	59.4	
Well	12	18.8	
R category			
R0	45	70.3	
R1/R2	19	29.7	
T-stage			
T1	2	3.1	
T2	11	17.2	
T3	38	59.4	
T4	13	20.3	
N-stage			
N0	26	40.6	
N1	38	59.4	
M-stage			
M0	58	90.6	
M1	6	9.4	
Overall pathological stage			
I	1	1.6	
II	24	37.5	
III	33	51.6	
IV	6	9.4	

### Quantitative polymerase chain reaction

RNA was extracted from cultured cells and clinical specimens using TRIzol reagent (Invitrogen), according to manufacturer’s instructions. Reverse transcription and quantitative polymerase chain reaction (qPCR) were performed as described [26–28]. In brief, 500 ng cDNA was reverse transcribed into cDNA using SuperScript III First-Strand Synthesis System for RT-PCR kit (Invitrogen), following manufacturer’s protocol. cDNA was then subjected for qPCR using Platinum Quantitative PCR SuperMix-UDG w/ROX (Invitrogen) and survivin-specific primers (forward: 5′-AAG GAC CAC CGC ATC TCT AC-3′ and reverse: 5′-CAG CTC CTT GAA GCA GAA GAA- 3′), for which this pair of primers were able to detect all splice variants of survivin. In parallel experiment, β-actin was used as an internal control for normalization and its specific primers were used (forward: 5′-CCA TCA TGA AGT GTG ACG TG-3′ and reverse: 5′-ATC CAC ATC TGC TGG AAG GT-3′). qPCR was performed in ABI PRISM 7700 Sequence Detection System (Applied Biosystems, Carlsbad, CA).

### Immunoblot

Proteins were extracted from cultured cells using ice-cooled Cell Lysis Buffer (Cell Signaling Technology, Danvers, MA). 30 μg protein was resolved onto a 12 % sodium dodecyl sulfate (SDS) polyacrylamide gel before subjected to electro-transfer onto the polyvinylidene fluoride (PVDF) membrane as described [29, 30]. After blocking with 5 % non-fat milk at room temperature for 2 h, membranes were incubated overnight with rabbit monoclonal antibody against survivin (Cell Signaling Technology, clone 71G4B7E, at 1:1,500 dilution) at 4 °C. This antibody was able to recognize all isoforms of survivin. Detection of β-actin as a loading control was achieved using β-actin antibody (Sigma-Aldrich, St Louis, MO, USA clone AC-74, at 1:10,000 dilution). Horseradish peroxidase (HRP)-conjugated secondary antibodies were used for recognizing primary antibodies, and the signals were subsequently viewed using ECL Plus Western blotting reagent pack (GE Healthcare Biosciences, Piscataway, NJ, USA) followed by autoradiography.

### Immunohistochemistry

Immunohistochemistry was performed as previously described [31–33]. Paraffin-embedded clinical sections were deparaffinized and rehydrated in xylene and alcohol. Antigen retrieval was performed by heating the sections in 0.1 mol/L citrus buffer (pH 6.0) in a microwave for 10 min. After quenching the endogenous peroxidase activities using 3 % hydrogen peroxide for 20 min at room temperature, the sections were incubated with 10 % normal goat serum for 1 h at room temperature to block the non-specific binding sites. After that, the sections were incubated with survivin antibody (1:100) at 4 °C overnight. Anti-rabbit labeled polymer-HRP provided in EnVision + System-HRP (Dako, Glostrup, Denmark) was used to detect survivin antibody and the incubation at room temperature lasted for 45 min. Finally, the signals were visualized by incubating sections with liquid DAB + (diaminobenzidine) (Dako) before counterstaining those sections with hematoxylin. A colon carcinoma specimen was used as a positive control. The substitution of survivin antibody with normal goat serum was treated as the negative control. The results were evaluated by Dr. KW Chan, a qualified pathologist, under light microscope. For each section, the expression of survivin was categorized according to the percentage of cancer cells stained positive with survivin antibody (with score from 0 to 4; 0 ≤ 5 %, 1 = 6–25 %, 2 = 26–50 %, 3 = 51–75 %, 4 ≥ 75 %) and the intensities of the signals (1+, 2+ and 3+). Then, a weighted index score (0–12) for each specimen was calculated by multiplying the values of these two categories [34], such that the tumors were segregated into two groups based on the expression of survivin, that is, low expression group (weighted index score 0–6) and high expression group (weighted index score 7–12).

### Statistical analysis

Data in the bar chart were expressed as mean ± SEM. Either Pearson’s chi-squared test or Fisher’s exact test, where appropriate, was used to assess the correlations for clinical analysis and immunohistochemistry. Kaplan–Meier method was employed for survival analysis, and the differences in survival were estimated using log-rank test. A *p* value of less than 0.05 was considered statistically significant. All the statistical analyzes were performed using SPSS 12.0 for Windows (Chicago, IL, USA).

## Results

### High expression of survivin in ESCC tissues and cell lines

**Fig. 1:**
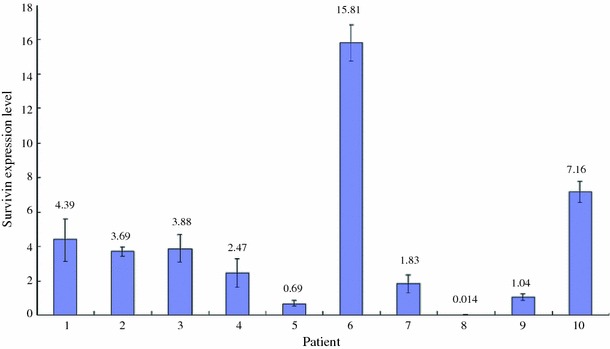
High mRNA expression of survivin in esophageal squamous cell carcinoma (ESCC) specimens. Quantitative polymerase chain reaction (qPCR) was performed to examine the mRNA expression of survivin in ESCC tissues and their adjacent non-tumor tissues. High expression of survivin mRNA was found in 7 of 10 ESCC tissues. The relative expression level of each non-tumor tissue was arbitrarily set at 1. The fold ratio of tumor versus non-tumor tissues is indicated above the *bar* for each patient. Experiment for each sample was repeated twice

**Fig. 2:**
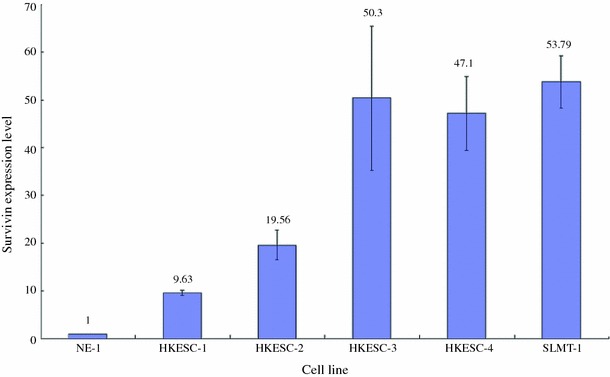
High mRNA expression of survivin in esophageal squamous cell carcinoma (ESCC) cell lines. Quantitative polymerase chain reaction (qPCR) was performed to examine the mRNA expression of survivin in 5 ESCC cell lines (HKESC-1, HKESC-2, HKESC-3, HKESC-4, and SLMT-1) and a non-neoplastic esophageal epithelial cell line NE-1. The relative expression of survivin in ESCC cell lines was compared to the NE-1 cell line and is represented as a relative fold ratio. High expression of survivin mRNA was associated with ESCC cells when compared to the NE-1 cells. The fold ratio of NE-1 cell line is arbitrarily set to 1 and the fold ratio of each ESCC cell line versus NE-1 cell line is indicated above the *bar* for each cell line. Each sample was performed in at least duplicate

**Fig. 3:**
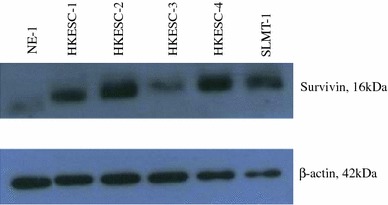
High protein level of survivin in esophageal squamous cell carcinoma (ESCC) cell lines. Western blot was performed to examine the protein level of survivin in ESCC cell lines (HKESC-1, HKESC-2, HKESC-3, HKESC-4, and SLMT-1) and a non-neoplastic esophageal epithelial cell line NE-1. High protein level of survivin was found in ESCC cells when compared to the NE-1 cells. This figure shows the representative image from several experiments

Up-regulation of survivin mRNA was demonstrated in 70 % (7 of 10) ESCC tissues when compared to their adjacent non-tumor tissues. The increase in expression ranged from ~twofold (patient 7) to ~16-fold (patient 6). ESCC tissues from patient 5 and 8 showed down-regulation of survivin mRNA compared with the adjacent non-tumor tissues. ESCC tissue from patient 9 had no apparent difference in the expression of survivin (Fig. 1). Five human ESCC cell lines and a non-neoplastic esophageal epithelial cell line were used to study the expression of survivin. The mRNA expression of survivin in all ESCC cell lines (HKESC-1, HKESC-2, HKESC-3, HKESC-4, and SLMT-1) was higher than that of non-neoplastic NE-1 cell line. In particular, HKESC-3, HKESC-4, and SLMT-1 cells demonstrated ~50-fold increase in survivin mRNA when compared to NE-1 cells (Fig. 2). Similar observation was found in the protein level of survivin in this panel of cell lines, such that high expression of survivin protein was observed in ESCC cells when compared to NE-1 cells having non-detectable level of survivin (Fig. 3).

### High nuclear survivin indicates ESCC patients in early-stage and without nodal metastasis

**Fig. 4:**
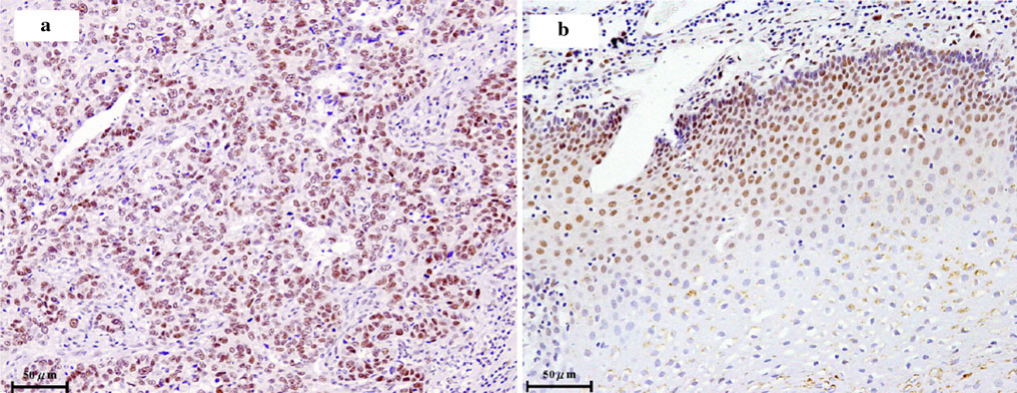
Nuclear localization of survivin in esophageal squamous cell carcinoma (ESCC) and non-tumor tissues. Immunohistochemistry was performed to study the localization of survivin in tumor (**a**) (*n* = 64) and non-tumor (**b**) (*n* = 10) tissues of ESCC patients. Intense staining of survivin was found in the cell nucleus of each specimen studied. Representative images are shown here. Original magnification, ×200

Immunohistochemistry was performed to reveal the localization of survivin in 64 tumor tissues of ESCC patients and 10 randomly selected adjacent non-tumor tissues from the same patient cohort. Positive control experiment showed staining of nuclear survivin, while negative control had no staining of survivin (data not shown). For both tumor and non-tumor tissues, survivin was found at the cell nuclei in all specimens examined (Fig. 4). Nuclear survivin was found in majority of cancer cells in ESCC tissues (Fig. 4a), while it was associated preferentially in the proliferating layer of the epithelium and not found in the upper and more differentiated layer in the non-tumor tissues (Fig. 4b).

When we correlated the expression of nuclear survivin with different clinicopathological parameters of ESCC patients, nuclear expression of survivin was inversely associated with the likelihood of developing nodal metastasis (*p* = 0.021). ESCC patients having high expression of nuclear survivin were less likely to develop nodal metastasis when compared to those patients with low expression of nuclear survivin. Nuclear survivin was also significantly associated with early-stage ESCC (*p* = 0.039), which means ESCC patients with high level of nuclear survivin were more likely to be in the early-stage of disease (Table 2). In addition to tumor stage, R category of resection is another prognostic parameter for ESCC patients; however, no correlation was found between the expression of nuclear survivin and this factor. ESCC patients having high or low expression of nuclear survivin had a median survival of 17.90 months (95 % CI: 4.67–31.13 months) and 14.75 months (95 % CI: 12.84–16.67 months), respectively. There was no statistically significant difference (*p* = 0.339) in the survival of patients having different expression of nuclear survivin (data not shown).

**Table 2:** Correlation of nuclear survivin with various clinicopathological parameters in esophageal squamous cell carcinoma patients

Clinicopathological parameters	Subgroups	Low expression of nuclear survivin	High expression of nuclear survivin	*p* value
Age (grouped)	Below or equal 65	14	17	0.617
	Above 65	18	15	
Gender	Female	9	8	1.000
	Male	23	24	
Smoking	Non smoker	15	16	1.000
	Smoker	17	16	
Level of tumor	Upper	5	5	0.346
	Middle	18	19	
	Lower	6	8	
	Double	3	0	
Tumor differentiation	Poor	8	6	0.822
	Moderate	18	20	
	Well	6	6	
R category	R0	25	20	0.274
	R1/R2	7	12	
T-stage	Early (T1/T2)	5	8	0.536
	Advanced (T3/T4)	27	24	
N-stage	N0	8	18	0.021
	N1	24	14	
M-stage	M0	27	31	0.196
	M1a/M1b	5	1	
Overall pathological stage	Early (stage I/II)	8	17	0.039
	Advanced (stage III/IV)	24	15	

## Discussion

This is the first report showing the correlations between nuclear survivin expression with nodal metastasis and pathological stage in ESCC patients. ESCC patients with high expression of nuclear survivin are mostly in early-stage of disease without nodal metastasis. In the literatures, there are conflicting results in the prognostic significance of survivin in ESCC. This may partly due to the different experimental methods used like immunohistochemistry and qPCR [2, 3, 35–40]. For instance, immunohistochemical studies have demonstrated significant correlation between nuclear survivin and poor prognosis of ESCC patients [35, 37]. On the contrary, Warnecke-Eberz and colleagues have suggested survivin mRNA as a favorable marker for ESCC based on their results derived from transcriptional studies [39]. However, there has been no immunohistochemical study thus far reporting on any positive effect of nuclear survivin in ESCC patients.

In early findings, survivin expression is connected to microvessel density [16]. Tumors with high capacity of microvessel have been shown to have high metastatic potential [41, 42]. In this report, high nuclear survivin expression may suggest retaining of survivin protein inside the nucleus, diminishing the effect of microvessel formation in the tumor and thus relating to the less likelihood of nodal metastases in ESCC.

Survivin is proposed to inhibit apoptosis after its phosphorylation by p34^cdc2^/cyclin B complex, while a survivin mutant (with mutation at alanine, T34A), which is not able to undergo phosphorylation, can induce apoptosis probably by substrate competition [4]. As different post-translational modifications could affect epitope accessibility of nuclear and cytoplasmic survivin, it is postulated that only cytoplasmic survivin can associate with and be phosphorylated by p34^cdc2^/cyclin B complex. On the other hand, nuclear survivin that remains unphosphorylated even in the presence of p34^cdc2^/cyclin B complex always favors and triggers apoptosis. Therefore, this helps to explain the different prognostic implications of cytoplasmic and nuclear survivin in cancers because of their different roles. Cells expressing survivin with mutation at nuclear exportation signal (NES) accumulated in the nucleus, and these cells demonstrated reduced cytoprotective capabilities because of the inability of these survivin mutants in protecting cells against X-irradiation and TNF-related apoptosis-inducing ligand (TRAIL)-induced apoptosis [5]. It is obvious that nuclear survivin has impaired cytoprotective functions [6, 7].

The survivin antibody we used in this study can recognize all splice variants of survivin, including survivin-2b for which this variant has proven pro-apoptotic effect by acting as a natural antagonist of anti-apoptotic survivin in tumor cells [8, 43, 44]. Suga et al. [45] found that colorectal carcinoma patients having a higher mRNA expression ratio of survivin-2b/wild-type survivin were associated with better prognosis and in early-stage disease, while patients with a lower expression of this ratio had shorter survival and advanced stage disease. Regarding the cellular level of survivin-2b, its expression was higher in less aggressive neuroblastoma and benign brain tumors when compared to their corresponding more aggressive types [46, 47]. Furthermore, Mahotka et al. [48] also observed a significant decrease in survivin-2b level during the progression of renal cell carcinoma. All these findings prove for the inhibitory role of survivin-2b in cancer development. Indeed, the cellular localizations of survivin-2b among different cell types are still under debate [44]. It is believed that the positive stains of nuclear survivin in ESCC tissues may also encompass survivin-2b, which is probably responsible for pro-apoptotic process that is observed in less aggressive tumor condition. Future work should be done to focus on studying the presence of survivin-2b in ESCC tissues.

In conclusion, we have shown for the first time in this study that up-regulation of nuclear survivin correlated with earlier pathological stage and less likelihood of nodal metastases in ESCC. This may be helpful in further dissecting the debate regarding the role of survivin as a prognostic marker in ESCC.
